# Supplementary material for: A generalized multipath delayed-choice experiment on a large-scale quantum nanophotonic chip
Source: Nat Commun. 2021 May 7;12:2712. doi: 10.1038/s41467-021-22887-6 (PMC8105384; doi:10.1038/s41467-021-22887-6)
Supplement: Supplementary file 1 — Supplementary Information [file 41467_2021_22887_MOESM1_ESM.pdf]

# Supplementary Information:

# A generalised multipath delayed-choice experiment on a large-scale quantum nanophotonic chip

Xiaojiong Chen[1,†], Yaohao Deng[1,†], Shuheng Liu[1,†], Tanumoy Pramanik[1,2], Jun Mao[1], Jueming Bao[1], Chonghao Zhai[1], Tianxiang Dai[1], Huihong Yuan[1], Jiajie Guo[1], Shao-Ming Fei[3], Marcus Huber[4,5], Bo Tang[6], Yan Yang[6,⋆], Zhihua Li[6], Qiongyi He[1,2,7,8,9,⋆], Qihuang Gong[1,2,7,8,9⋆], Jianwei Wang[1,2,7,8,9⋆]

[1] *State Key Laboratory for Mesoscopic Physics, School of Physics, Peking University, Beijing, 100871, China*
[2] *Beijing Academy of Quantum Information Sciences, Beijing 100193, China*
[3] *School of Mathematical Sciences, Capital Normal University, Beijing 100037, China*
[4] *Institute for Quantum Optics and Quantum Information – IQOQI Vienna, Austrian Academy of Sciences, Boltzmanngasse 3, 1090 Vienna, Austria*
[5] *Vienna Center for Quantum Science and Technology, Atominstitut, TU Wien, 1020 Vienna, Austria*
[6] *Institute of Microelectronics, Chinese Academy of Sciences, Beijing 100029, China*
[7] *Frontiers Science Center for Nano-optoelectronics & Collaborative Innovation Center of Quantum Matter, Peking University, Beijing, 100871, China*
[8] *Collaborative Innovation Center of Extreme Optics, Shanxi University, Taiyuan 030006, Shanxi, China*
[9] *Peking University Yangtze Delta Institute of Optoelectronics, Nantong 226010, Jiangsu, China.*
[†] *These authors contributed equally to this work.*
⋆ *emails: yyang10@ime.ac.cn, qiongyihe@pku.edu.cn, qhgong@pku.edu.cn, jww@pku.edu.cn*

## 1 Supplementary Note 1: Device characterization

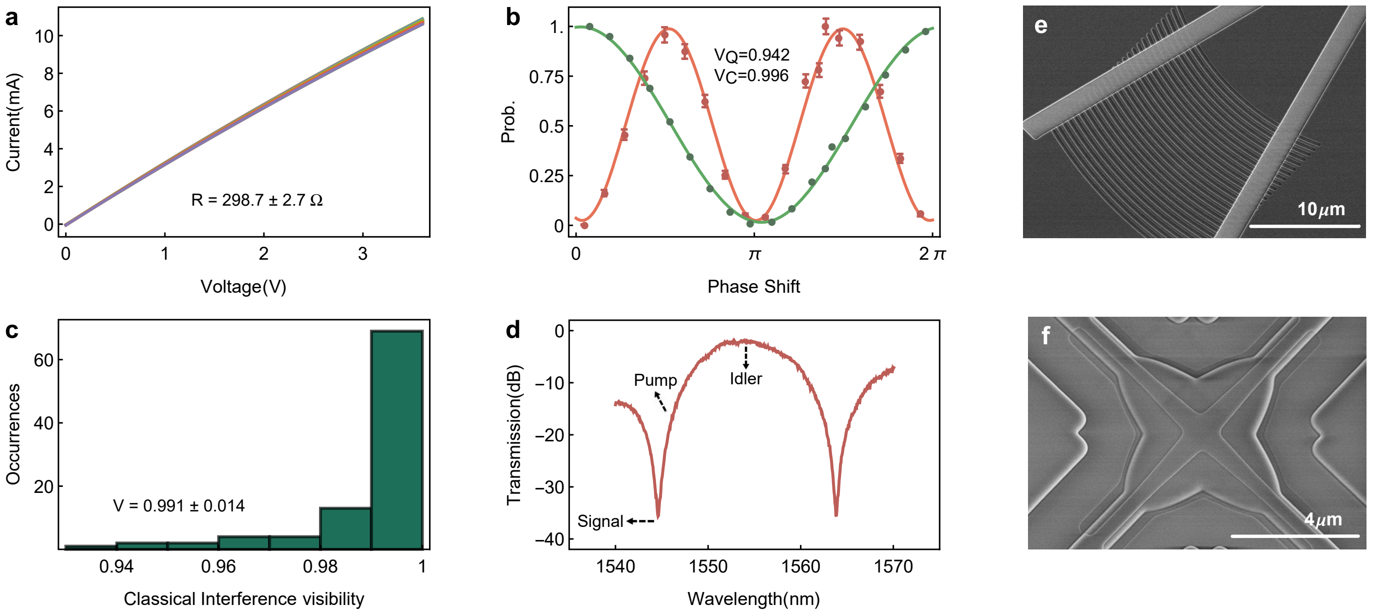


**Supplementary Figure 1 Characterizations of integrated photonic components.** **a,** Current-voltage characterization of all 95 thermo-optic phase-shifters. The resistance ($R$) of phase-shifter was measured to be $(298.7 \pm 2.7)\Omega$. The fabricated phase-shifters exhibit high uniformity of performance. **b,** Single-photon classical interference fringe (green) and two-photon quantum interference fringe (red). High visibilities are quantified. Error bars in plot are estimated from photon Poissonian statistics. **c,** A histogram for all measured contrast visibilities (in total 95), characterized from the classical interference in MZIs. The visibility ($V$) of 0.991 with $\pm 1\sigma$ of 0.014 was obtained. **d,** Measured transmission spectrum for an asymmetric MZI. The spectrum is normalized to a straight waveguide with grating couplers. The wavelength for pump, signal and idlers photons are indicated by arrows, respectively. The asymmetric MZIs allow us to separate the created signal photons and idler photons. **e** and **f,** Scanning electron microscope (SEM) images for fabricated grating coupler and waveguide crosser, respectively, that are both double-layer etched structures, as shown in Fig.1h.

The single-mode silicon waveguides were fabricated with a cross size of 500 nm $\times$ 220 nm (see SEM image in Fig.1g). The spiral SFWM sources were designed with a length of 1.4cm, allowing efficient generation of entangled photons. Multimode interferometers (MMIs) that owns large fabrication tolerance were used as 50:50 balanced beamsplitters. The manipulation of single photons relies on the thermal-optic

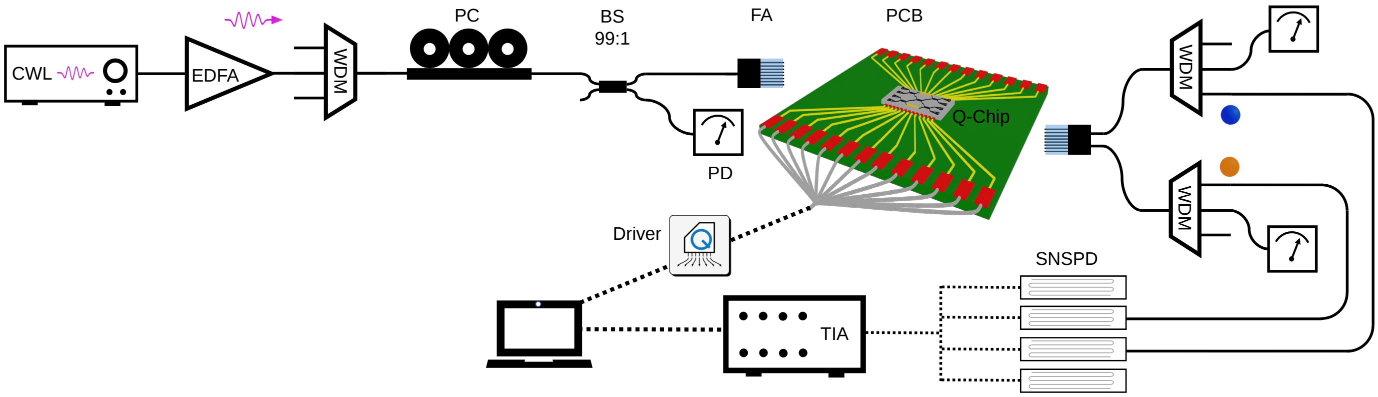


**Supplementary Figure 2 Experimental setup.** A continuous-wave laser (CWL) with a wavelength of $\lambda_p = 1550.11$ nm was amplified to 40 mW by an erbium doped fiber amplifier (EDFA). The bright laser was used as the pump source for the SFWM photon-pair generation. To remove the amplified spontaneous emission noise from the pump light, we applied a wavelength-division multiplexing (WDM) filter on the pump light. The WDM owns a bandwidth of 1 nm, channel space of 1.6 nm and extinction ratio of more than 100 dB. A fiber-based polarization controller (PC) was used to optimize the polarization before the chip, ensuring the TE (transverse electric) mode input. A pair of path-coded entangled photons with different wavelengths, signal photon at $\lambda_s = 1545.31$ nm and idler photon at $\lambda_i = 1554.91$ nm, were generated via the SFWM process. The photons were manipulated and measured on-chip, by controlling the thermo-optic phase-shifters. All phase-shifters were individually accessed and driven by a multichannel voltage driver, with a 16-bit resolution and $\mu$s response time. The photons were then coupled off the chip into an optical fiber array (FA) for photon detection. Before that, the residual pump photon were removed from the signal and idler photons by two DWDMs, positioned after the chip. The residual pump were also monitored by photodiodes (PDs) for the stabilization of the system. The two single photons were ultimately detected by an array of superconducting nanowire single-photon detectors (SNSPDs), with a 85% detection efficiency and 65ps time jitter. The two-fold photon coincidences were recored by a time interval analyzer (TIA). The silicon quantum chip was packaged and wired bonded on a PCB (see an optical microscope image in Fig.1f). A temperature stabilization system is equipped to reduce the thermal cross-talk between heaters. CWL: continuous-wave laser; EDFA: erbium doped fiber amplifier; WDM: wavelength-division multiplexing; PC: polarization controller; BS: beam splitter; PD: photo-diodes; FA: fiber array; PCB: Printed circuit board; SNSPD: superconducting nanowire single-photon detector; TIA: time interval analyzer. Solid lines are optical fibers, and dotted lines are electric wires.

tuning of phase-shifters by TiN heaters. The phase-shifters were designed with a 100 µm-length and 3 µm-width. Supplementary Figure 1a shows the current-voltage characterization of all 95 thermo-optic phase-shifters, showing a resistance of $R = (298.7 \pm 2.7)\Omega$. Supplementary Figure 1b shows the single-photon classical interference (green) and two-photon quantum interference (red), having a visibility of 0.996 and 0.942, respectively. The contrast visibility is defined as $(N_{\text{max}} - N_{\text{min}})/(N_{\text{max}} + N_{\text{min}}))$, where $N$ denotes the number of measured photons. The characterization of all 2-path MZIs was performed by measuring the classical interference. A histogram of all measured contrast classical visibilities is shown in Supplementary Fig.1c, reporting a measured visibility of 0.991 with $\pm 1\sigma$ of 0.014. We used $d(d-1)$ 2-MZIs to realize the $d$-BS1 and $d$-BS2, which are fully reconfigurable for the choice of different dimension $d$. In our experiment, we used asymmetric MZIs as on-chip filters to separate the generated signal photon at $\lambda_s = 1545.31$ nm and idler photon at $\lambda_i = 1554.91$ nm. The asymmetric MZI filters were designed with a free spectral range (FSR) of $\lambda_{\text{FSR}} = 19.2$ nm (see Supplementary Fig.1d), in order to match the FSR of off-chip WDM filters as shown in Supplementary Fig.2. The 70-nm shallowly etched grating couplers (see SEM image in Supplementary Fig.1e) in the focused configuration enabled a coupling efficiency of about 40%, that allowed us to couple photons in and out of the chip. The insertion loss of waveguide crossers (see SEM image in Supplementary Fig.1f) was measured to be below $-0.2$ dB and crosstalk well below $-35$ dB. The chip was packaged on a printed circuit board (PCB), and implemented in the experimental setup, as shown in Supplementary Fig 2.

Note that photon-pairs can be also created in the remaining parts of the chip after the spiral sources. However, these photons contributed nearly negligible to all of our measurements of multipath wave-particle transition and duality relation. This is because that we measured the two photon coincidences between the detectors $D_8$ and $D_0$-$D_7$, and this coincidence measurement excludes the counting of the extra photons which were created after the spiral source. One may argue that the photons created in the latter parts may bring in accidental coincidences. In order to suppress such accidental coincidences as much as possible, we expanded the connecting and routing waveguides to a width of $5\mu m$ to weaken the unwanted SFMW effect. The adoption of on-chip asymmetric MZIs (see Fig.1 in main text and Supplementary Fig.1d) can remove 50% of the residual pump, thus reducing the generation of accidental coincidences. All these processes ensure small influence of our measurements, which was confirmed by the observations of high quantity two-photon interference (Supplementary Fig.1b), high fidelity topographically reconstructed density matrix (Supplementary Fig.3), as well as high fidelity wave-particle transitions. In future, the uses of pump rejection filter[1] or optical resonator source[2] can help further suppress background noises in the whole process.

## 2 Supplementary Note 2: Measurement of high-order interference in the $d$-path integrated interferometer

Double-path or double-slit experiments have allowed successful tests of Bohr's duality rule[3–6]. In the multipath interferometric experiment, it is of fundamental significance to rule out the existence of high-order interference terms[7]. Ruling out of the presence of such high-order interference is the basis of testing the multipath wave-particle duality.

We measured the magnitude of high-order interference term with respect to the second-order interference term (mutual coherence term). As an example, we characterized the fourth-order interference, by reconfiguring our device as a 4-path interferometer. The control photon was projected into the $|1\rangle$ basis, which functions as a heralding single photon in this measurement, and indicates the presence of the target

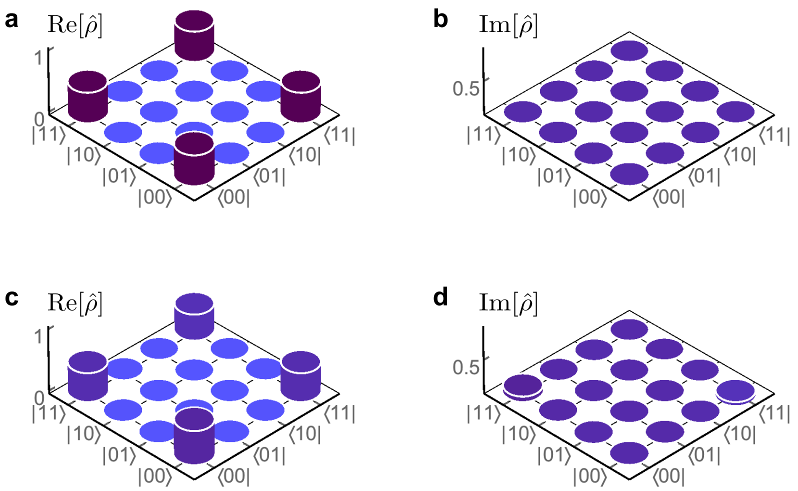


**Supplementary Figure 3 Experimental quantum state tomography. a,** Real and **b,** imaginary part of the ideal maximally entangled state $(|00\rangle + |11\rangle)/\sqrt{2}$. **c,** Real and **d,** imaginary part of the experimentally reconstructed density matrix. In our experiment, over-complete tomography techniques with 36 measurements in total were used to reconstruct the state. Quantum state fidelity of $F_Q = 0.962 \pm 0.002$ was measured, which is defined as $\langle\phi|\hat{\rho}|\phi\rangle$, where $\hat{\rho}$ represents the reconstructed state in **(c** and **d)** and $|\phi\rangle$ is the ideal Bell state in **(a** and **b)**.

photon. The target photon was sent through the 4-path MZI for the test of high-order interference (the four paths are labelled as 1, 2, 3 and 4, respectively). In particular, we set the $\alpha$ phase of the control photon to be $\pi$, that corresponds to the full-wave case. In this regard, we looked into the most obvious case of wave interference. And, we collected the data at the prime maxima of the fringe when the $\theta$ phase was set as $\pi$ (see Fig.3), that led to the minimal noises from photons fluctuations.

The second-order interference term (mutual coherence term) $I_{II}(ij)$ is represented as[8]:

$$I_{II}(ij) = P_{II}(ij) - P_I(i) - P_I(j), \tag{1}$$

where $P_I(i)$ ($P_I(j)$) refers to the measured number of photons (i.e, the measured two-fold coincidence counts) when only the $i$-path ($j$-path) remains open; $P_{II}(ij)$ is the measured number of photons when both the $i$- and $j$-path are open; the subscripts of $I, II$ denote the number of opened modes.

The fourth-order interference term $I_{IV}(1234)$ can be described as[7]:

$$I_{IV}(1234) = P_{IV}(1234) - \sum_{i<j} P_{II}(ij) + 2\sum_{i} P_I(i), \tag{2}$$

where $P_{IV}(1234)$ denotes the measured number of photons when all of the 4 paths are open. All pairs of the $P_{II}(ij)$ terms, in total 6 terms, were measured by blocking all paths, but not the $i$ and $j$ paths. All of the $P_I(i)$ terms, in total 4 terms, were measured by blocking all paths, but not the $i$ path.

A normalized magnitude of $\kappa$ is adopted to represent the relative deviation from Born's rule. The $\kappa$ is defined as a ratio of the fourth-order interference term to the sum of all second-order interference terms[9]:

$$\kappa = \frac{I_{IV}(1234)}{\sum_{i<j} I_{II}(ij)}. \tag{3}$$

The $\kappa$ value is expected to be zero. However, it is reasonable to be bounded by a certain accuracy, due to the presence of noises and errors in the experiment. Our experimental measurement returns a $\kappa = -0.0031 \pm 0.0047$, as shown in Fig.2 in main text. This experimental result thus rules out the existence of the fourth-order interference within an accuracy of $10^{-3}$ bound, and lays the basis for the further analysis of multipath wave-particle duality.

In our experiment, the dominant error and noise came from the Poissonian fluctuation of single photons. Note that the dark counts have been subtracted for the estimation of $\kappa$; the nonlinear compensation of SNSPDs was not taken into account in our measurements. Another main error was introduced by the non-perfect 2-path MZIs used in our integrated nanophotonic chip. Each of 2-path MZIs provides about 30 dB extinction ratio (see Supplementary Fig1.c). This means that we could not completely turn on/off the paths in the current integrated optic device. For example, in order to block the certain paths (for the measurement of $P_{IV}$, $P_{II}$, and $P_I$), we have to route away the photons by switching off the corresponding 2-path MZIs embedded in the $d$-BSs (see Fig.1e). There are about 0.1% possibility of photon leakage between neighboring paths, and such leakage could bring in noises and errors in the measurement of high-order interference. The device is among one of the largest quantum photonic devices with 95 thermal-optical phase-shifters. Though temperature stabilisation and liquid cooling were applied in our system, thermal crosstalk is still in presence. Better management and control of thermal crosstalk are required for

integrated quantum photonics especially when the quantum devices scale up to the very large-scale. Other contributions might come from the instability of our experimental setup, such as intensity fluctuation of the pump laser, and fiber-chip decoupling during the measurement.

## 3 Supplementary Note 3: Multipath wave-particle quantum superposition and classical mixture

In this section we discuss the state evolution throughout the device, and derive probability distributions for the quantum superposition and classical mixture cases. In the next section, Sec.4, we discuss the delayed-choice of measurement apparatus, and through the choice of measurement we drive the multipath duality relation.

Photon-pair are created at the spiraled sources that are 1.4 cm-long deep etching waveguides, based on the spontaneous four-wave mixing (SFWM) nonlinear process. The dispersion of the waveguides are engineered to efficiently create photon pairs near 1550nm. Two SFWM sources are coherently pumped to generate a bipartite state:

$$c_0|1\rangle_{i,0}|1\rangle_{s,0}|0\rangle_{i,1}|0\rangle_{s,1} + c_1|0\rangle_{i,0}|0\rangle_{s,0}|1\rangle_{i,1}|1\rangle_{s,1}, \tag{4}$$

where $|1\rangle_i$ ($|1\rangle_s$) indicates the photon number state of the idler (signal) photon being in its 0-th or 1-th spatial mode (subscripts); $|0\rangle_i$ ($|0\rangle_s$) indicates the vacuum state; $c_{0,1}$ represents the complex amplitude in each mode, having $|c_0|^2 + |c_1|^2 = 1$. The two non-degenerate photons generated by SFWM are deterministically separated using asymmetric on-chip MZI filters and swapped by a waveguide crosser (see Fig.

$$c_0|0\rangle_i|0\rangle_s + c_1|1\rangle_i|1\rangle_s, \tag{5}$$

where the coefficients $c_{0,1}$ can be chosen by arbitrary controlling the pump distribution and its phase. Maximally path-entangled Bell states $(|0\rangle_i|0\rangle_s + e^{i\delta}|1\rangle_i|1\rangle_s)/\sqrt{2}$ can be obtained with a uniform excitation of the sources. For simplicity, we rewrite it as:

$$(|0\rangle_C|0\rangle_T + |1\rangle_C|1\rangle_T)/\sqrt{2}, \tag{6}$$

where the signal photon plays as the target while the idler photon plays as the control photon. We locally manipulate the state of each qubit by a SU(2) operation consisting of a MZI with an additional phase-shifter (see Fig.1). Then, the target photon passes through two processes coherently, the particle-process (open $d$-path MZI, without $d$-BS2) and the wave-process (closed $d$-path MZI, with $d$-BS2), see Fig.1e.

The choice of which-process the photon takes, either the wave or particle process, is entangled with the state of the control photon. Note that both processes the photon take are completely identical, except that of the $d$-BS2.

$$\frac{1}{\sqrt{2}}(|0\rangle_C|\mathrm{P}\rangle_T + |1\rangle_C|\mathrm{W}\rangle_T), \tag{7}$$

$$|\mathrm{P}\rangle_T = \frac{1}{\sqrt{d}}\sum_{m=0}^{d-1} e^{i\theta_m}|m\rangle_T, \quad |\mathrm{W}\rangle_T = \frac{1}{\sqrt{d}}\sum_{m=0}^{d-1}\sum_{k=0}^{d-1} h_{mk}^{(d)} e^{i\theta_k}|m\rangle_T, \tag{8}$$

where $|\mathrm{P}\rangle_T$ and $|\mathrm{W}\rangle_T$ indicate the state of target photon after passing the particle process or the wave process; $|m\rangle$ refers to the logical state in the $k$-th path. An arbitrary local rotation $\{\alpha, \delta\}$ is applied to the control photon, then the state evolves into:

$$\frac{|0\rangle_C\left(\sin\frac{\alpha}{2}|\mathrm{P}\rangle_T + e^{i\delta}\cos\frac{\alpha}{2}|\mathrm{W}\rangle_T\right) + |1\rangle_C\left(\cos\frac{\alpha}{2}|\mathrm{P}\rangle_T - e^{i\delta}\sin\frac{\alpha}{2}|\mathrm{W}\rangle_T\right)}{\sqrt{2}}, \tag{9}$$

where $\{\alpha, \delta\}$ represent the $\{\sigma_y, \sigma_z\}$ rotations of the control photon. When we project the control photon in to the basis of $|1\rangle_C$, the state of the target photon is reduced to:

$$|\psi\rangle_T = \cos\frac{\alpha}{2}|\mathrm{P}\rangle_T - e^{i\delta}\sin\frac{\alpha}{2}|\mathrm{W}\rangle_T. \tag{10}$$

Note that, up to this step, the two processes, *i.e.* wave and particle processes, are classical distinguishable. A $d$-mode quantum erasure consisted by an array of $d$ number of 2-BSs, is ultimately adopted to erase which-process information and ensure that the wave and particle processes are in a coherent superposition as:

$$|\psi\rangle_T = \frac{1}{\sqrt{2}}\left[\left(\cos\frac{\alpha}{2}|\mathrm{P}\rangle_{\mathrm{up}} - ie^{i\delta}\sin\frac{\alpha}{2}|\mathrm{W}\rangle_{\mathrm{up}}\right) + \left(i\cos\frac{\alpha}{2}|\mathrm{P}\rangle_{\mathrm{below}} - e^{i\delta}\sin\frac{\alpha}{2}|\mathrm{W}\rangle_{\mathrm{below}}\right)\right], \tag{11}$$

where up (below) refers to the upper (below) mode of the $d$-mode quantum eraser, which are denoted at the position of $\mathrm{D}_i$ ($\mathrm{D}_i'$) in Fig.1e. This ultimately results in the state-process entanglement. This approach of state-process entanglement has been adopted for the implementations of controlled-unitary gate for quantum simulations[10] and for the double-path delayed-choice experiments[11].

### 3.1 Quantum superposition of wave and particle

When considering one of the two output modes, say up mode, then the state of target photon is:

$$|\psi\rangle_T = \frac{1}{\sqrt{N}}\left(\cos\frac{\alpha}{2}|\mathrm{P}\rangle_{\mathrm{up}} - ie^{i\delta}\sin\frac{\alpha}{2}|\mathrm{W}\rangle_{\mathrm{up}}\right), \tag{12}$$

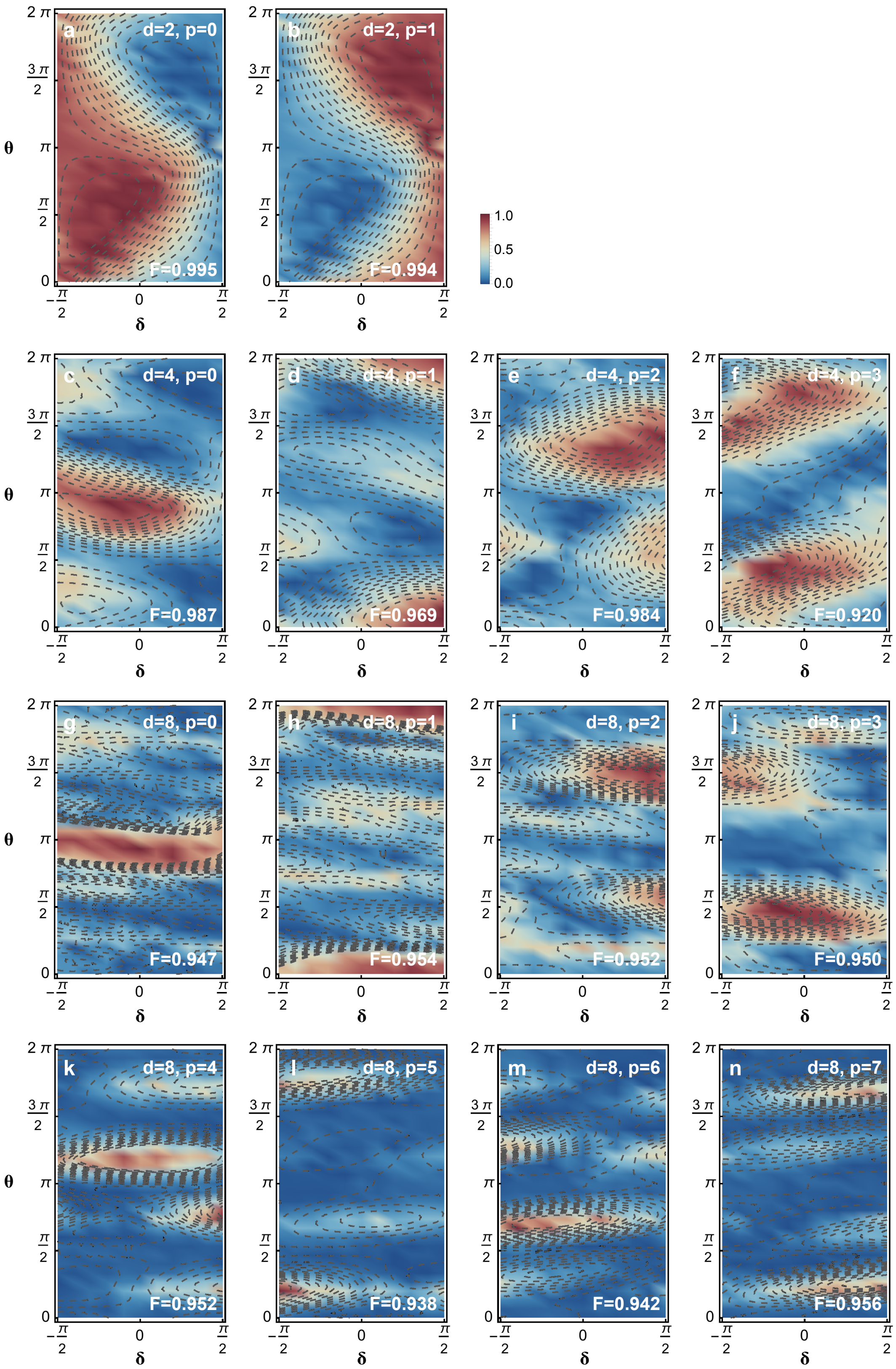


**Supplementary Figure 4 Multipath quantum wave-particle transition measured at the $D_i$ mode,** $i \in [0, d-1]$**.** The wave-particle transitions are measured in the context of genuine quantum-superposition case having $\alpha = 3\pi/2$, when the particle and wave nature are in maximal superposition. **a** and **b,** for $d = 2$; **c-f,** for $d = 4$; **g-n,** for $d = 8$. The $p$ indicates the number of output port, and $d$ is the number of paths. The $\delta$-dependence of interference patterns confirms the existence of genuine wave-particle superposition. The $p = 0$ data in **(a)**, **(c)** and **(g)** are shown in Fig.3, which are provided for comparison. Density distributions (colored) represent experimental data, while contour lines (dashed) represent theoretical results. The $F$ denotes the classical fidelity, and hgh fidelities are obtained for all measurements.

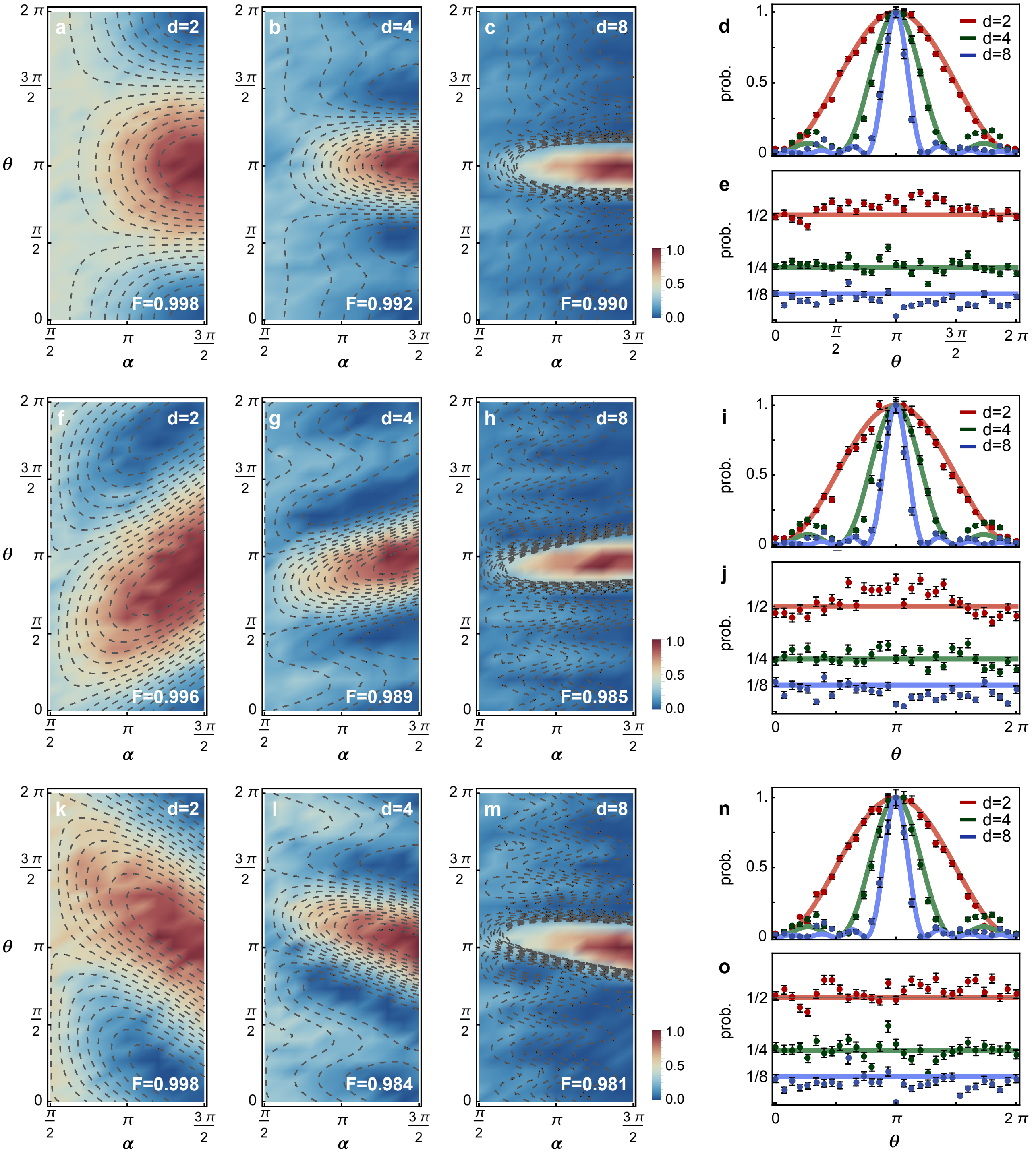


**Supplementary Figure 5 Experimental results of delayed-choice multipath wave-particle transition measured in the $\sigma_x$-basis.** All measured data here are collected at the complementary basis $\sigma_x$, which is to demonstrate the existence of entanglement that enables the quantum delayed-choice experiment. Note the internal phase $\delta$ is chosen as zero here. Measured transitions between the particle and wave properties in three different scenarios: **a-c,** classical-mixture; **f-h,** quantum-superposition upper mode; **k-m,** quantum-superposition down mode. They are quantified by probability distributions (normalized coincidences) for different $\alpha$ of the control and $\theta_d$ of the target [$\theta_d = k(\theta - \pi)$ was chosen], in the 2, 4 and 8-path experiments. Density distributions (colored) represent experimental data, while contour lines (dashed) represent theoretical results. The $F$ denotes the classical fidelity $\sum_i \sqrt{p_i q_i}$ summing over the whole space of $(\theta, \alpha)$ or $(\theta, \delta)$, where $p_i$ and $q_i$ are the measured and theoretical probabilities. High fidelities are obtained for all measurements. Results in **(a-c)** are consistent with classical optical multi-slit interference. The asymmetry of transition patterns in the quantum case **(f-h)** and **(k-m)** stem from the interference of wave and particle states. **d-e,** classical observations, **i-j** and **n-o,** quantum observations of full wave nature at $\alpha = \pi$, and full particle nature at $\alpha = 0$, for $d$ =2, 4 and 8. The interference fringe becomes sharper for $d$-path interference, while it yields multi-level quantization from $d$-outcomes of which-path information. Points represent experimental data, while lines represent theoretical values. All error bars ($\pm 3\sigma$) are estimated from photon Poissonian statistics.

where $N$ is a normalization coefficient. Note that Supplementary Equation 12 represents the quantum superposition of wave and particle properties. We obtain the probability of detecting the target photon in the $m$-th upper mode (after the erasure process):

$$I_{m,\text{quantum}}(\hat{\theta}_d, \alpha, \delta) = \frac{1}{N}\left|\frac{1}{\sqrt{d}}\cos\frac{\alpha}{2} - ie^{i\delta}\frac{1}{\sqrt{d}}\sum_{k=0}^{d-1} h_{mk}^{(d)} e^{i\theta_k}\sin\frac{\alpha}{2}\right|^2. \tag{13}$$

Relying on the observation of no high-order interference, the cross terms in Supplementary Equation 13 indicate the interference of wave and particle processes. For ease of representation, we choose the $d$-mode phase-shifters to be $\theta_k = k(\theta - \pi)$, with $k = 0, ...d-1$; however any setting of $\{\theta_k\}_{k=0}^{d-1}$ is available. As an example, we consider the probability distribution of the first port, upper mode, *i.e.* detected at $D_0$:

$$I_{0,\text{quantum}}(\hat{\theta}_d, \alpha, \delta) = \frac{1}{N}\left|\frac{1}{\sqrt{d}}\cos\frac{\alpha}{2} - ie^{i\delta}\frac{e^{id(\theta-\pi)} - 1}{d(e^{i(\theta-\pi)} - 1)}\sin\frac{\alpha}{2}\right|^2, \tag{14}$$

with $N = 1 + \frac{\sin\alpha\sin\delta\sin d(\theta-\pi)}{d^{3/2}\sin(\theta-\pi)}$, which is dependent on the configuration of the control photon's $\{\alpha, \delta\}$ state. In the experiment, the probability $I_{0,\text{quantum}}$ is obtained by the normalization of two-fold coincidence, over that of all output ports.

### 3.2 Classical mixture of wave and particle

When considering both the upper and below parts of the $d$-mode eraser and classically mix their outcomes, then the result is equivalent to the case that the which-process information is not erased and the two processes remain classical distinguishable. This is also equivalent to tracing out the control qubit. The system thus turns into the classical mixture of wave and particle:

$$\rho_T = \cos^2\frac{\alpha}{2}|\text{P}\rangle\langle\text{P}| + \sin^2\frac{\alpha}{2}|\text{W}\rangle\langle\text{W}|. \tag{15}$$

The probability of detecting the target photon in the $m$-th port of eraser is given by:

$$I_{m,\text{classical}}(\hat{\theta}_d, \alpha) = \frac{1}{d}\cos^2\frac{\alpha}{2} + \frac{1}{d}\left|\sum_{k=0}^{d-1} h_{mk}^{(d)} e^{i\theta_k}\right|^2\sin^2\frac{\alpha}{2}. \tag{16}$$

In contrast to the quantum superposition case in Supplementary Note 3.1, the probability distribution in the classical case is $\delta$-independent as it stems from the classical mixture. The cross terms that represent the interference between wave and particle characters in the quantum case (Supplementary Equation 13), are cancelled out – the probability distribution $I_{m,\text{classical}}$ is a classical mixture of wave and particle properties. Choose the same $\theta_k = k(\theta - \pi)$, with $k = 0, ...d-1$, and consider the probability distribution of the first port (sum of the upper and down mode), we have:

$$I_{0,\text{classical}}(\hat{\theta}_d, \alpha) = \frac{1}{d}\cos^2\frac{\alpha}{2} + \left|\frac{e^{id(\theta-\pi)} - 1}{d(e^{i(\theta-\pi)} - 1)}\right|^2\sin^2\frac{\alpha}{2}. \tag{17}$$

Experimental results for both wave-particle quantum superposition and classical mixture, are shown in Fig.3 in main text (measured in the computational $\sigma_z$ basis) and Supplementary Fig.5 (measured in the complementary $\sigma_x$ basis). Supplementary Fig. 4 shows the measured probability distributions for other ports. The experimental results are in good agreement with theoretical results, confirmed by the estimation of high classical fidelity.

## 4 Supplementary Note 4: generalised multipath duality relation in the delayed-choice experiment

In this section, we represent how to construct the quantum coherence $\mathcal{C}_d$ and path-distinguishability $\mathcal{D}_d$ in theory, and how to measure them in experiment. We discuss two different scenarios, wave-particle classical mixture and quantum superposition.

Take the state of target photon after the $d$-BS1 as the initial state. We consider the maximal coherent state as:

$$|\Psi\rangle_0 = \frac{1}{\sqrt{d}}\sum_{k=0}^{d-1}|k\rangle, \tag{18}$$

where $\{|k\rangle\}_{k=0}^{d-1}$ is the logical basis that defines the reference frame. $\hat{\theta}_d$ is applied on $|\Psi\rangle_0$. For simplicity, phases from the $d$-BS1 operator can be absorbed by the $\hat{\theta}_d$. To measure the genuine duality property of the target photon, not in a too naive way, the wave-particle measurement $\hat{\text{M}}_m$ is determined in a delayed manner, which means the state of $d$-BS2 is determined in a delayed manner, by the state of $\{\alpha, \delta\}$. The choice of measurement $\hat{\text{M}}_m(\alpha, \delta)$ is a posterior one, projecting the state either into the full wave, full particle or their superposition basis.

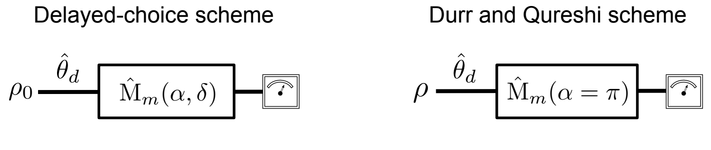

Our delayed-choice scheme is a reverse process of Durr's original scheme for measuring the $l_2$-norm coherence[12] and Qureshi's scheme for measuring the $l_1$-norm coherence[13]. In Durr's and Qureshi's scheme: given the state $\rho$, the goal is to estimate the coherence from interference patterns. This is allowed by performing the wave measurement $\hat{M}_m(\alpha=\pi)$ on the $\rho$. The $\hat{M}_m(\alpha=\pi)$ is corresponding to the balanced $d$-BS operator. The probability that the photon projects to $|\Psi\rangle_0$ is given by:

$$I=\frac{1}{d}\sum_{j=0}^{d-1}\sum_{k=0}^{d-1}\rho_{jk}e^{i\left(\theta_j-\theta_k\right)}=\frac{1}{d}\left(\sum_{j=0}^{d-1}\rho_{jj}+\sum_{j\neq k}\left|\rho_{jk}\right|\cos\left(\theta_j-\theta_k+\arg\rho_{jk}\right)\right)=\frac{1}{d}\left(1+\sum_{k\neq j}\left|\rho_{jk}\right|\cos\left(\theta_j-\theta_k+\arg\rho_{jk}\right)\right). \tag{19}$$

Properly choosing the $\theta_{i,j}$ phases and compensating for $\arg\rho_{jk}$, the primary maxima $I_{\max}=(1+\sum_{j\neq k}|\rho_{jk}|)/d$ can be obtained. The generalised visibility defined as $\mathcal{V}_d=\frac{1}{d-1}$

$$\mathcal{V}_d=\mathcal{C}_d=\frac{1}{d-1}\sum_{j\neq k}\left|\rho_{jk}\right|, \tag{20}$$

where $\mathcal{C}_d$ is the normalized $l_1$-norm coherence[14].

In our experiment, the duality relation is to be formalized in the context of delayed-choice way. Our scheme is a Hermitian conjugation of Durr's scheme, which are in fact equivalent when we measure the probability distribution. This allows us to adopt Durr's and Qureshi's framework to quantify the coherence as well as the duality relation. We will discuss in detail for both classical and quantum cases as below.

### 4.1 Duality relation in the quantum superposition scenario

The probability of detecting photon at the $m$-th port and upper mode, that is obtained by normalizing the measured coincidences from the detectors$\{D_8^{'},D_m\}$, is given by:

$$I_{m,\text{quantum}}=\langle m|\hat{\theta}_d\rho_0\hat{\theta}_d^{\dagger}|m\rangle, \tag{21}$$

where $\rho_0=|\Psi\rangle_{00}\langle\Psi|$, $\hat{\theta}_d$ is the phase operator; $|m\rangle$ is the wave-particle basis which is defined by the state of $d$-BS2,

$$\hat{O}=\cos\frac{\alpha}{2}\hat{I}-ie^{i\delta}\sin\frac{\alpha}{2}\hat{H}_d, \tag{22}$$

which is a superposition of present and absent state, controlled by $\{\alpha,\delta\}$. Note that the "absence" term ($\cos\frac{\alpha}{2}\hat{I}$) represents the measurement in the particle basis, while the "presence" term ($\sin\frac{\alpha}{2}\hat{H}_d$) represents the measurement in the wave basis. The state of $d$-BS2 $\hat{O}$ allows the rotation of projective measurement between the wave and particle bases, such that the maximal coherence state $|\Psi\rangle_0$ is measured in the $|m\rangle$ basis defined as:

$$|m\rangle=\frac{1}{\sqrt{N_d}}\sum_{k=0}^{d-1}(\Delta_{(m-k)}\cos\frac{\alpha}{2}+ie^{-i\delta}h_{mk}^{(d)}\sin\frac{\alpha}{2})|k\rangle, \tag{23}$$

where $N_d=1+\sin\delta\sin\alpha/\sqrt{d}$ is the normalization coefficient; where $\Delta_x$ refers to the Kronecker function, $\Delta_x=1$ for $x=0$ and $\Delta_x=0$ for $x\neq 0$; $h_{mk}^{(d)}=\frac{1}{\sqrt{d}}(-1)^{m\odot k}$. At the first port upper mode, the projective basis is explicitly given by:

$$|m,0\rangle=\frac{1}{\sqrt{N_d}}\begin{pmatrix}\cos\frac{\alpha}{2}+\frac{ie^{-i\delta}}{\sqrt{d}}\sin\frac{\alpha}{2}\\ \frac{ie^{-i\delta}}{\sqrt{d}}\sin\frac{\alpha}{2}\\ \vdots\\ \frac{ie^{-i\delta}}{\sqrt{d}}\sin\frac{\alpha}{2}\end{pmatrix} \tag{24}$$

The probability can be calculated by $I_{0,\text{quantum}}=\text{Tr}\left[\hat{M}_0\hat{\theta}_d\rho_0\hat{\theta}_d^{\dagger}\right]$, where $\hat{M}_0=|m,0\rangle\langle m,0|$. This results in the same probability distribution as Supplementary Equation 14. Basically, our delayed-choice scheme is the Hermitian conjugation of Durr's scheme. Revealing either the wave or particle nature is dependent on which complementary measurement to perform. That means, measurement apparatus plays the same role as the intrinsic duality of the photon. Both the photon and measurement apparatus have to be taken into account. In another words, the observation of duality nature of photons is reference dependent. The $d$-BS2 operator $\hat{O}$ transforms the reference-frame from the logical basis $|k\rangle$ into the wave-particle basis $|m\rangle$. For ease of representation, we denote $\rho$ to represent the density matrix for the entire system having the photon and delayed-choice measurement.

#### 4.1.1 Coherence $\mathcal{C}_d$

The normalized $l_1$-norm coherence is given by[14]:

$$\mathcal{C}_d=\frac{1}{d-1}\sum_{j\neq k}\left|\rho_{jk}\right|, \tag{25}$$

which is believed to be a good measure of wave nature[15,16]. We aim to obtain the coherence $\mathcal{C}_d$ from interference patterns. The coincidence between detectors $\{D_8^{'}, D_0\}$ is normalized to obtain the probability distribution:

$$I_{0,\text{quantum}} = \frac{1}{d}\sum_{j,k=0}^{d-1}\rho_{jk}e^{i(\theta_j-\theta_k)} = \frac{1}{d}\left(\sum_{j=0}^{d-1}\rho_{jj} + \sum_{j\neq k}\left|\rho_{jk}\right|\cos\left(\theta_j-\theta_k+\arg\rho_{jk}\right)\right). \tag{26}$$

Note only the mutual coherence terms are present in Supplementary Equation 26. The $I_{0,\text{quantum}}$ equals to Supplementary Equation 19. Choose a proper setting of $\hat{\theta}_d$ and compensate for the phases of off-diagonal elements:

$$\theta_0 = -\arctan\frac{\cos\frac{\alpha}{2}\cos\delta}{\frac{1}{\sqrt{d}}\sin\frac{\alpha}{2}+\cos\frac{\alpha}{2}\sin\delta} \qquad \theta_1=\cdots=\theta_{d-1}=0, \tag{27}$$

we get the prime maxima:

$$I_{\max} = \frac{1}{d}\left(\sum_{j=0}^{d-1}\rho_{jj}+\sum_{j\neq k}\left|\rho_{jk}\right|\right) = \frac{1}{N_d d}\left(\sqrt{\frac{1}{d}\sin^2\frac{\alpha}{2}+\cos^2\frac{\alpha}{2}+\frac{1}{\sqrt{d}}\sin\delta\sin\alpha}+\frac{d-1}{\sqrt{d}}\sin\frac{\alpha}{2}\right)^2. \tag{28}$$

Note that, the prime maxima ($I_{\max}$) of interference pattern consists of two parts: off-diagonal terms, representing coherence of the target photon, and diagonal terms, representing the incoherent term that have no contribution to interference $\sum_{j=0}^{d-1}\left|\rho_{jj}\right| = 1$. The incoherent term is defined as:

$$I_{\text{inc}} = \frac{1}{d}\sum_{j=0}^{d-1}\rho_{jj}, \tag{29}$$

where

$$\rho_{00} = \frac{1}{N_d}\left(\frac{1}{d}\sin^2\frac{\alpha}{2}+\cos^2\frac{\alpha}{2}+\frac{1}{\sqrt{d}}\sin\delta\sin\alpha\right), \quad \rho_{11}=\cdots=\rho_{d-1d-1}=\frac{1}{N_d d}\sin^2\frac{\alpha}{2}. \tag{30}$$

In our experiment, the $\rho_{ii}$ were measured as following: only the $i$ path remains open and all the other $d-1$ paths were blocked. Then the coincidences between detectors $\{D_8^{'}, D_i\}$ were measured. We measured the coincidences from all single-opening paths, and averaged them to obtain the normalized probability.

The generalised visibility $\mathcal{V}_d$ is defined by the disparity of primary maxima $I_{\max}$ and the incoherent term $I_{\text{inc}}$[13].

$$\mathcal{V}_d = \frac{1}{d-1}\frac{I_{\max}-I_{\text{inc}}}{I_{\text{inc}}}. \tag{31}$$

Substituting Supplementary Equations 28, 29 and 30 into Supplementary Equation 31, we obtain

$$\mathcal{V}_d = \frac{1}{d-1}\sum_{j\neq k}\left|\rho_{jk}\right| = \mathcal{C}_d. \tag{32}$$

Thus, we prove that the generalised visibility defined above is equivalent to the normalized $l_1$-norm coherence, *i.e.* $\mathcal{V}_d = \mathcal{C}_d$. The result of Supplementary Equation 32 derived in our multipath delayed-choice system is the same as the one derived by Qureshi[13]. Supplementary Figure 6 reports the measured $l_1$-norm coherence for the $d$-path interference fringes ($d \in [2,8]$), when our device is implemented in the delayed-choice wave-particle quantum superposition.

### 4.1.2 Distinguishability $\mathcal{D}_d$

The measurement of which-path information is posteriori in our context of delayed-choice scenario. In our scheme, path-distinguishability is introduced after target photon entering the interferometer and adjusted by the state of quantum controlled $d$-BS2 – so as the delayed choice of measurement apparatus, thus the which-path information we obtain is indeed posteriori[17].

We adopted the path-distinguishability $\mathcal{D}_d$ in[12,18,19] to measure which-path information:

$$\mathcal{D}_d = \sqrt{1-\left(\frac{1}{d-1}\sum_{i\neq j}\sqrt{\rho_{ii}\rho_{jj}}\right)^2}, \tag{33}$$

which is basically a generalisation of $d=2$ distinguishability. To experimentally measure the $\mathcal{D}_d$, we basically measured the diagonal terms as we have discribed above.

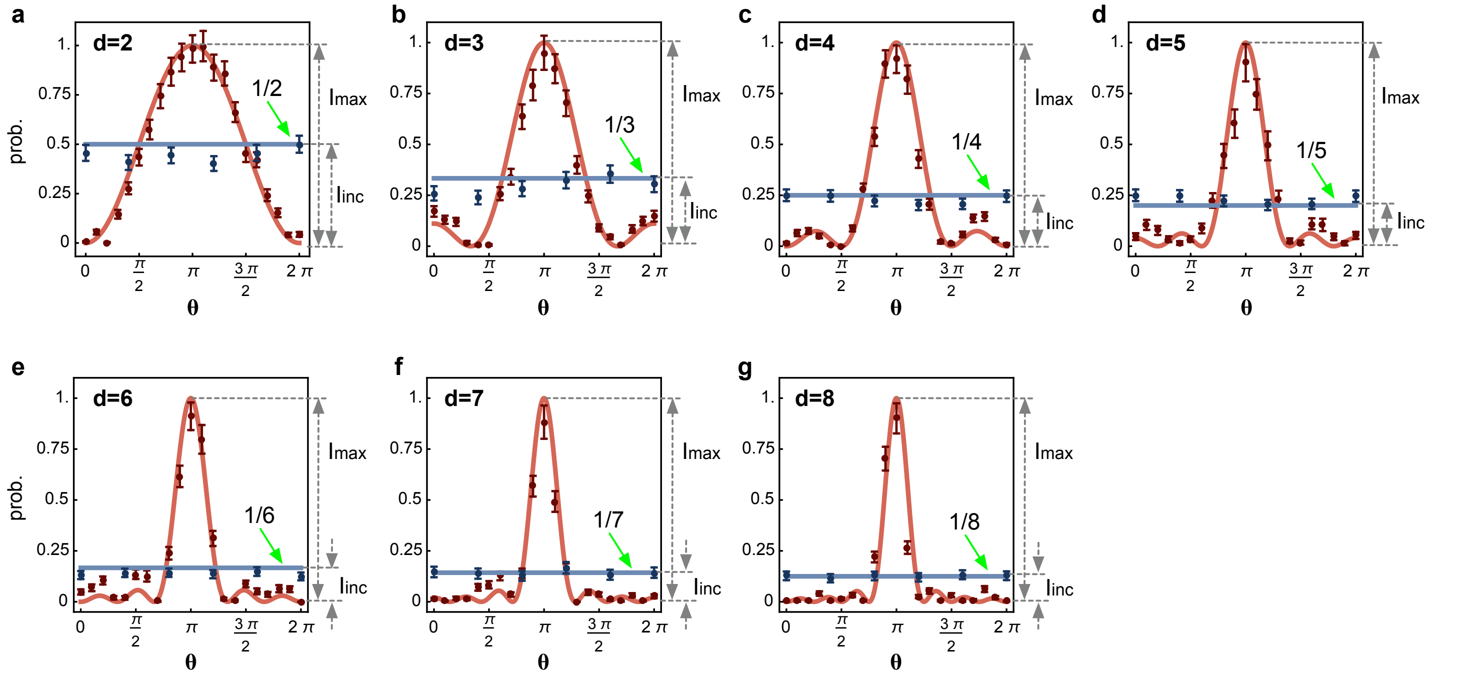


**Supplementary Figure 6 Measurement of generalised visibility for $d$-path quantum interference fringes when $\alpha = \pi$.** We consider the $d$-path full-wave quantum interference fringes (red) and incoherent terms (blue) for **a,** $d = 2$; **b,** $d = 3$; **c,** $d = 4$; **d,** $d = 5$; **e,** $d = 6$; **f,** $d = 7$; **g,** $d = 8$. When $\alpha = \pi$, there is nothing to do with the interference of the two properties, so that the quantum fringes agree with classical ones. The generalised visibility $\mathcal{V}_d$ is determined by the disparity of the primary maxima $I_{\max}$ and incoherent term $I_{\text{inc}}$. $I_{\text{inc}}$ was obtained by recording the probability of $i$-outcomes only having the $i$-path open, and the mean of measured $I_{\text{inc}}$ was used to estimate the $\mathcal{V}_d$. The $\mathcal{V}_d$ is equivalent to the quantum coherence $\mathcal{C}_d$, an amount of coherence information can be directly probed from interference patterns, with no need to probing the full density matrix. The $l_1$ norm coherence $\widetilde{\mathcal{C}}_{l1}$ for $d$-path is shown in Fig.5a. In all above plots, points represent experimental data, while lines represent theoretical values. All error bars ($\pm\sigma$) are estimated from photon Poissonian statistics.

### 4.1.3 Duality relation

Given all the definitions introduced above, we now obtain the explicit forms of $\mathcal{C}_d$ and $\mathcal{D}_d$:

$$\mathcal{C}_d = \frac{(d-2)\sin^2\frac{\alpha}{2} + 2\sin\frac{\alpha}{2}\sqrt{1+(d-1)\cos^2\frac{\alpha}{2} + \sqrt{d}\sin\delta\sin\alpha}}{d+\sqrt{d}\sin\delta\sin\alpha}, \tag{34}$$

$$\mathcal{D}_d = \sqrt{1 - \left(\frac{(d-2)\sin^2\frac{\alpha}{2} + 2\sin\frac{\alpha}{2}\sqrt{1+(d-1)\cos^2\frac{\alpha}{2} + \sqrt{d}\sin\delta\sin\alpha}}{d+\sqrt{d}\sin\delta\sin\alpha}\right)^2}. \tag{35}$$

Bohr's duality relation for the $d$-path interferometer can thus be formalized as:

$$\mathcal{C}_d^2 + \mathcal{D}_d^2 \leq 1. \tag{36}$$

Importantly, as $|\rho_{jk}| = \sqrt{|\rho_{jj}\rho_{kk}|}$, the inequality always saturates (*i.e.* $\mathcal{C}_d^2 + \mathcal{D}_d^2 = 1$) in the context of wave-particle quantum superposition. In the next Section 4.2 for the case of wave-particle classical mixture, we will see that we cannot achieve the upper bound and the equality breaks up at $\alpha \neq \{0, \pi\}$, resulting in $\mathcal{C}_d^2 + \mathcal{D}_d^2 < 1$.

## 4.2 Duality relation in the classical mixture scenario

Now we turn to the classical case, where the interference between wave and particle process is cancelled out. We consider the probability distribution of $m$-th port, in experiment by normalizing the coincidences between detectors $\{\mathrm{D}_8', \mathrm{D}_m$ and $\mathrm{D}_m'\}$:

$$I_{m,\text{classical}} = \frac{\langle m|\hat{\theta}_d\rho_0\hat{\theta}_d^\dagger|m\rangle + \langle m'|\hat{\theta}_d\rho_0\hat{\theta}_d^\dagger|m'\rangle}{2}, \tag{37}$$

where $|m\rangle$ and $|m'\rangle$ are two wave-particle basis at the upper and bottom modes of the $m$-measurement. We consider the measurement at the first port:

$$|m,0\rangle = \begin{pmatrix} \cos\frac{\alpha}{2} + \frac{ie^{-i\delta}}{\sqrt{d}}\sin\frac{\alpha}{2} \\ \frac{ie^{-i\delta}}{\sqrt{d}}\sin\frac{\alpha}{2} \\ \vdots \\ \frac{ie^{-i\delta}}{\sqrt{d}}\sin\frac{\alpha}{2} \end{pmatrix} \qquad |m',0\rangle = \begin{pmatrix} -i\cos\frac{\alpha}{2} - \frac{e^{-i\delta}}{\sqrt{d}}\sin\frac{\alpha}{2} \\ -\frac{e^{-i\delta}}{\sqrt{d}}\sin\frac{\alpha}{2} \\ \vdots \\ -\frac{e^{-i\delta}}{\sqrt{d}}\sin\frac{\alpha}{2} \end{pmatrix}, \tag{38}$$

and mark $\hat{\mathrm{M}}_0 = (|m,0\rangle\langle m,0| + |m',0\rangle\langle m',0|)/2$, and $I_{0,\mathrm{classical}} = \mathrm{Tr}\left[\hat{\mathrm{M}}_0\hat{\theta}_d\rho_0\hat{\theta}_d^{\dagger}\right]$. The explicit form of probability is given in Supplementary Equation 17. Similar to the quantum case, the $\rho$ represents the density matrix for the whole system with the target photon and choice of measurement.

#### 4.2.1 Coherence $\mathcal{C}_d$

The explicit form of $I_{0,\mathrm{classical}}$ is rewritten into:

$$I_{0,\mathrm{classical}} = \frac{1}{d}\sum_{j,k=0}^{d-1}\rho_{jk}e^{i(\theta_j-\theta_k)} = \frac{1}{d}\left(\sum_{j=0}^{d-1}\rho_{jj} + \sum_{j\neq k}\left|\rho_{jk}\right|\cos\left(\theta_j-\theta_k+\arg\rho_{jk}\right)\right). \tag{39}$$

In contrast to the quantum case, here $\arg\rho_{jk}=0$. To get the prime maxima, we set $\hat{\theta}_d$ to be:

$$\theta_k = 0 \qquad for \;\; k = 0 \ldots d-1. \tag{40}$$

The coincidence between detectors $\{\mathrm{D}_8^{'}, \mathrm{D}_0 \text{ and } \mathrm{D}_0^{'}\}$ is normalized to obtain the probability distribution, and we obtain the prime maxima:

$$I_{\max} = \frac{1}{d}(\sum_{j=0}^{d-1}\rho_{jj} + \sum_{j\neq k}\left|\rho_{jk}\right|) = \sin^2\frac{\alpha}{2} + \frac{1}{d}\cos^2\frac{\alpha}{2}. \tag{41}$$

The diagonal terms are given by:

$$\rho_{00} = \frac{1}{d}\sin^2\frac{\alpha}{2} + \cos^2\frac{\alpha}{2}, \quad \rho_{11} = \cdots = \rho_{d-1d-1} = \frac{1}{d}\sin^2\frac{\alpha}{2}. \tag{42}$$

As stated in Section 4.1, the normalized $l_1$-norm coherence is given by the disparity of primary maxima $I_{\max}$ and the incoherent term $I_{\mathrm{inc}}$, see Supplementary Equation 31. Substituting Supplementary Equations 41 and 42 into Supplementary Equation 31, we obtain:

$$\mathcal{V}_d = \frac{1}{d-1}\frac{I_{\max}-I_{\mathrm{inc}}}{I_{\mathrm{inc}}} = \sin^2\frac{\alpha}{2} = \mathcal{C}_d. \tag{43}$$

Note that $\mathcal{C}_d$ is independent of $d$.

#### 4.2.2 Distinguishability $\mathcal{D}_d$

Given the diagonal terms we have obtained in Supplementary Equation 42, and substituting Supplementary Equation 42 into Supplementary Equation 33, we obtain the path-distinguishability $\mathcal{D}_d$ for the classical case:

$$\mathcal{D}_d = \sqrt{1-\left(\frac{d-2}{d}\sin^2\frac{\alpha}{2} + \frac{2}{d}\sin\frac{\alpha}{2}\sqrt{1+(d-1)\cos^2\frac{\alpha}{2}}\right)^2}. \tag{44}$$

#### 4.2.3 Duality relation

Given the explicit form of $\mathcal{C}_d$ and $\mathcal{D}_d$ for the classical case in Supplementary Equations 43 and 44, the duality relation for $d$-path interferometer is formalized as:

$$\mathcal{C}_d^2 + \mathcal{D}_d^2 \leq 1. \tag{45}$$

The inequality in the classical case saturates ($\mathcal{C}_d^2 + \mathcal{D}_d^2 = 1$) only at $\alpha = \{0,\pi\}$, while it cannot achieve the upper bound and the equality breaks up at $\alpha \neq \{0,\pi\}$, resulting in $\mathcal{C}_d^2 + \mathcal{D}_d^2 < 1$. Experimental results for the classical duality relation are shown in Fig.4. Note that when $d=2$ the duality equation $\mathcal{C}_d + \mathcal{D}_d = 1$ is satisfied (see ref. [11]), but not for the generalised one with $d > 2$.

*Lemma* **Pure state is the sufficient and necessary condition for duality equality $\mathcal{C}_d^2 + \mathcal{D}_d^2 = 1$.**

**Proof** From the main text, it's easy to conclude that pure state is a sufficient condition.

If $\mathcal{C}_d^2 + \mathcal{D}_d^2 = 1$, $|\rho_{kl}| = \sqrt{\rho_{kk}\rho_{ll}}$ is satisfied for any $(k, l)$ pair. For generality, we write state $\rho$ as $\rho = \lambda_i \rho^{(i)}$, where $\lambda \neq 0$ and all $\rho^{(i)}$ are pure states. We use Einstein summation convention here. Introduce symbols $\rho_{kl} = z$, $\rho_{kl}^{(i)} = z_i$, $\rho_{kk} = x$, $\rho_{kk}^{(i)} = x_i$, $\rho_{ll} = y$, $\rho_{ll}^{(i)} = y_i$. For pure states, $|z_i| = \sqrt{x_i y_i}$.

On the one hand, $|z| = |\lambda_i z_i| \leq \lambda_i |z_i|$. The equation holds when all $z_i = \rho_{kl}^{(i)}$ have the same angle in the complex plane. On the other hand, $\sqrt{xy} = \sqrt{(\lambda_i x_i)(\lambda_j y_j)} = \sqrt{\lambda_i \lambda_j x_i y_j}$. Obviously, $x_i y_j + x_j y_i \geq 2\sqrt{x_i x_j y_i y_j}$. Therefore, $\sqrt{xy} = \sqrt{\lambda_i \lambda_j x_i y_j} \geq \sqrt{\lambda_i \lambda_j \sqrt{x_i x_j y_i y_j}} = \sqrt{(\lambda_i \sqrt{x_i y_i})^2} = \lambda_i \sqrt{x_i y_i}$. The equation holds when $x_i y_j = x_j y_i$ for any $(i, j)$ pair. In other words, corresponding diagonal elements among $\rho^{(i)}$ are proportionable.

According to analysis above, $\sqrt{xy} \geq \lambda_i \sqrt{x_i y_i} = \lambda_i |z_i| \geq |z|$. Consider that these two equation conditions are satisfied for any pair of $(k, l)$, we can find that $\rho^{(i)}$ are the same. Therefore, state $\rho = \lambda_i \rho^{(i)}$ must be a pure state. Then pure state is also a necessary condition.

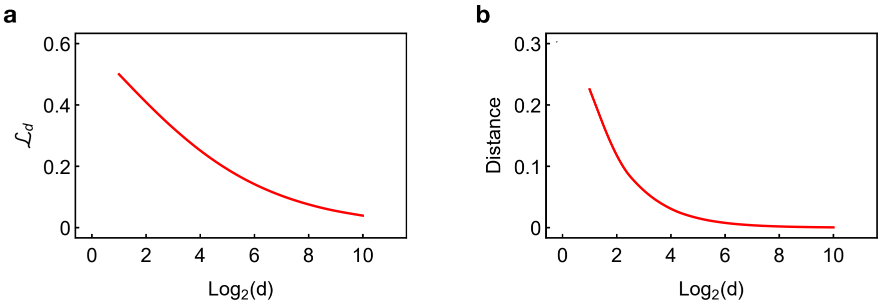


**Supplementary Figure 7 Characterization of lost information and Pearson distance in a large $d$-path interferometer. a,** The calculated $\mathcal{L}_d$ in the classical mixture case. $\mathcal{L}_d$ represents classical lack of knowledge of the quantum system. **b,** The calculated Pearson distance $d_{X,Y}$ between the quantum-superposition distribution and classical-mixture distribution. When the $d_{X,Y}$ approaches 0, the classical and quantum distributions tend to be same in the large $d$-path interferometer.

# 5 Supplementary Note 5: Wave-particle duality in large $d$-path interferometric experiments

In this section we discuss the difference between the wave-particle classical-mixture and quantum-superposition cases, in a $d$-path interferometer with a large number of modes. In the quantum superposition case, the pure states of target photon (given in Supplementary Equation 12) always saturates the duality relation of $\mathcal{C}_d^2 + \mathcal{D}_d^2 = 1$. For the classical mixed states given in Supplementary Equation 15, the equality breaks up at $\alpha \neq \{0, \pi\}$, *i.e.* $\mathcal{C}_d^2 + \mathcal{D}_d^2 < 1$. We define a quantity as $\mathcal{L}_d$ the loss of information:

$$\mathcal{L}_d = 1 - \mathcal{C}_d^2 - \mathcal{D}_d^2. \tag{46}$$

$\mathcal{L}_d = 0$ for pure states. The $\mathcal{L}_d$ value is caused by tracing out the control photon, that is entangled with the target photon passing through the system. In the classical mixture case, the value of $\mathcal{L}_d$ is minimal at the full-particle and full-wave points at $\alpha = \{0, \pi\}$, and maximal at certain setting of $\alpha$ (e.g, $\alpha = \pi/2$ for $d = 2$), due to the quantum correlation between the control photon and target photon. In Supplementary Fig.7a, the loss of information is getting lower for the larger $d$-path system. The value of $\mathcal{C}_d^2 + \mathcal{D}_d^2$ approaches to the unity, indicating smaller gap between the classical-mixture and quantum-superposition cases. Since the $l_1$-norm coherence $C_d$ for the classical case is independent of $d$ (see Supplementary Equation 43), for larger $d$, less loss of information $\mathcal{L}_d$ occurs due to the larger distinguishability of path-information $\mathcal{D}_d$ (see Supplementary Equation 44). This can be understood from Supplementary Equation 38 that in the larger $d$-MZI, the probability of detecting photons at the port $D_0$ becomes higher (when choosing the $\hat{\mathrm{M}}_0$ measurement), while the probabilities of other ports decrease, resulting in higher distinguishability of path-information.

We then adopt Pearson distance to quantitively describe the difference between the two distributions, that are the quantum-superposition distribution and classical-mixture distribution. Pearson distance is obtained by subtracting the Pearson correlation coefficient ($\rho_{X,Y}$) from 1,

$$d_{X,Y} = 1 - \rho_{X,Y}, \quad \rho_{X,Y} = \frac{\mathcal{E}\left[(X - \mu_X)(Y - \mu_Y)\right]}{\sigma_X \sigma_Y}, \tag{47}$$

where random variables $X$ and $Y$ represent the distributions of classical-mixture (e.g. Fig.3a-c) and quantum-superposition (e.g. Fig.3f-h), respectively; $\mathcal{E}$, $\mu$, $\sigma$ are the expectation, mean value and standard deviation. As shown in Supplementary Fig.7b, we find that the Pearson distance tends to 0 when the dimension $d$ increases. The two transition distributions behave more identical with increasing $d$. In this regard, the wave-particle quantum-superposition distribution returns to the classical-mixture distribution, in the large $d$-path interferometer.

# References


1. S. Paesani *et al.* Generation and sampling of quantum states of light in a silicon chip. *Nat. Phys.* **15,** 925–929 (2019).
2. D. Llewellyn *et al.* Chip-to-chip quantum teleportation and multi-photon entanglement in silicon. *Nat. Phys.* **16,** 148–153 (2020).

3. P Grangier, G Roger & A Aspect. Experimental evidence for a photon anticorrelation effect on a beam splitter: a new light on single-photon interferences. *Europhys Lett.* **1,** 173–179 (1986).
4. A. Zeilinger, R. Gähler, C. G. Shull, W. Treimer & W. Mampe. Single- and double-slit diffraction of neutrons. *Rev. Mod. Phys.* **60,** 1067–1073 (1988).
5. S. Dürr, T. Nonn & G. Rempe. Origin of quantum-mechanical complementarity probed by a 'which-way' experiment in an atom interferometer. *Nature* **395,** 33–37 (1998).
6. S. Gerlich *et al.* Quantum interference of large organic molecules. *Nat. Commun.* **2,** 263 (2011).
7. R. Sorkin. Quantum mechanics as quantum measure theory. *Mod. Phys. Lett. A* **09,** 3119–3127 (1994).
8. N. Bohr. The quantum postulate and the recent development of atomic theory. *Nature* **121,** 580–590 (1928).
9. U. Sinha, C. Couteau, T. Jennewein, R. Laflamme & G. Weihs. Ruling out multi-order interference in quantum mechanics. *Science* **329,** 418–421 (2010).
10. J. Wang *et al.* Experimental quantum Hamiltonian learning. *Nat. Phys.* **13,** 551–555 (2017).
11. F. Kaiser, T. Coudreau, P. Milman, D. B. Ostrowsky & S. Tanzilli. Entanglement-enabled delayed-choice experiment. *Science* **338,** 637–640 (2012).
12. S. Dürr. Quantitative wave-particle duality in multibeam interferometers. *Phys. Rev. A* **64,** 042113 (2001).
13. T. Paul & T. Qureshi. Measuring quantum coherence in multislit interference. *Phys. Rev. A* **95,** 042110 (2017).
14. T. Baumgratz, M. Cramer & M. B. Plenio. Quantifying coherence. *Phys. Rev. Lett.* **113,** 140401 (2014).
15. M. N. Bera, T. Qureshi, M. A. Siddiqui & A. K. Pati. Duality of quantum coherence and path distinguishability. *Phys. Rev. A* **92,** 012118 (2015).
16. E. Bagan, J. A. Bergou, S. S. Cottrell & M. Hillery. Relations between coherence and path information. *Phys. Rev. Lett.* **116,** 160406 (2016).
17. V. Jacques *et al.* Delayed-choice test of quantum complementarity with interfering single photons. *Phys. Rev. Lett.* **100,** 220402 (2008).
18. B. C. Hiesmayr & M. Huber. Multipartite entanglement measure for all discrete systems. *Phys. Rev. A* **78,** 012342 (2008).
19. P. Roy & T. Qureshi. Path predictability and quantum coherence in multi-slit interference. *Phys. Scr.* **94,** 095004 (2019).
